# Measuring self-control in a wild songbird using a spatial discounting task

**DOI:** 10.1007/s10071-024-01911-4

**Published:** 2024-10-25

**Authors:** Ella McCallum, Rachael C. Shaw

**Affiliations:** School of Biological Sciences, Te Herenga Waka Victoria University of Wellington, Wellington, New Zealand

**Keywords:** Self-control, Spatial discounting, Inhibitory control, Delay of gratification, Spatiotemporal choice, Intertemporal choice

## Abstract

Self-control allows animals to resist instant gratification and invest more time and/or energy in better outcomes. However, widespread temporal self-control tasks lack ecological validity for many species, and few studies have explored whether self-control can be measured in the wild. We used a spatial discounting task resembling natural foraging decisions to measure self-control in wild toutouwai (*Petroica longipes*), a songbird endemic to New Zealand. Birds chose between a near, low-quality food item and a high-quality food item further away. Toutouwai showed striking individual variation in their self-control abilities. Validation tests suggested that our task reliably measured self-control in a spatial foraging context. However, individual-level performance was confounded by food preferences and the satiation and/or learning effects associated with increasing trial number, limiting the applicability of this task as a measure of individual variation in self-control. Nonetheless, we found no correlation between an individual’s self-control and their inhibitory control measured using a detour task, suggesting that self-control is a distinct ability from the suppression of impulsive motor actions in toutouwai. This study demonstrates for the first time that a bird is capable of self-control in a spatial context and provides suggestions for how future researchers may robustly quantify individual differences in self-control in the wild.

**Supplementary Information:**

The online version contains supplementary material available at 10.1007/s10071-024-01911-4.

## Introduction

Animals must often choose between taking an immediately available option or investing more time and/or effort in obtaining a better one (Beran 2015). This ability, known as self-control, is thought to be important across a range of contexts, from foraging decisions to mate-selection (Stevens and Stephens 2008, 2010). Capacities for self-control differ across (Tobin and Logue 1994; Stevens et al. 2005a; Miller et al. 2019; Brucks et al. 2022b) and within species (Schnell et al. 2021, 2022; Brucks et al. 2022b, a), and researchers have long sought to understand the evolutionary causes and consequences of this variation. To date, however, self-control tasks have largely been restricted to the laboratory, which may bias performance due to greater environmental predictability and the absence of competitors and predators during testing (Fawcett et al. 2012; Kidd et al. 2013; Jackson et al. 2018). Additionally, the ecological validity of temporal discounting tasks used to assess self-control has been questioned, as these tasks seldom resemble natural foraging decisions (Stephens and Anderson 2001; Hayden 2016). To this end, an emerging challenge in self-control research is determining which tasks can provide robust, ecologically relevant measures of this ability in the wild.

Self-control has been almost exclusively examined in a temporal context with delay of gratification tasks. Here, subjects choose whether to forego an immediate reward in favour of a better one after a delay (Beran 2015; Miller et al. 2019). While some species, such as great apes (Dufour et al. 2007; Rosati et al. 2007), have shown good self-control in delay of gratification tasks, many other species behave comparatively impulsively (Rachlin and Green 1972; Ainslie 1974; Ramseyer et al. 2006; Vick et al. 2010). This impulsivity may represent a real preference for immediate rewards in some animals, for example because delayed rewards may be intercepted by competitor, or because waiting prevents animals from engaging in other activities (Fawcett et al. 2012; Mallpress 2021). Equally, however, classic laboratory-based delay of gratification paradigms may overestimate impulsivity because they lack ecological validity (Stephens and Anderson 2001; Hayden 2016). For example, waiting stationarily for rewards to become available may be intuitive to ambush predators, but not active foragers, which naturally move through space to locate food.

The importance of ecological validity in self-control testing has been shown in rhesus macaques (*Macaca mulatta*). Compared to a classic delay of gratification task, macaques showed better self-control in a task that mimicked natural foraging decisions by requiring animals to choose between remaining in a patch for diminishing rewards, or moving to a new patch to obtain delayed, but larger, rewards (Blanchard and Hayden 2015). An additional problem with delay of gratification tasks is that they can require close interaction with a human experimenter (Hillemann et al. 2014; Beran et al. 2016) or potentially fear inducing apparatuses (Logan et al. 2020; Brucks et al. 2022b). As a result, the use of these tasks is often restricted to human-habituated, captive animals. However, wild animals may prioritise immediate rewards more strongly than their captive counterparts as they experience greater food insecurity and environmental unpredictability, which are linked to worse self-control in humans (Kidd et al. 2013; Jackson et al. 2018). Delayed rewards may also be riskier in the wild as competitors and predators can prevent their collection (Fawcett et al. 2012; Mallpress 2021). For these reasons, measuring self-control in a natural setting using ecologically relevant tasks can improve the reliability of results.

Spatial discounting tasks may offer an ecologically valid way of measuring self-control in the wild. In these tasks, subjects can choose to take a nearer, lower-quality reward, or travel further for a higher-quality alternative (Stevens et al. 2005b). Obtaining the higher-quality reward thus requires both a time delay and increased effort. Spatial discounting tasks closely resemble the foraging decisions of mobile animals and may therefore be readily understood with little training (Mallpress 2021). By contrast, extensive training is often needed to teach subjects the rules in delay of gratification tasks, such that these tasks may inadvertently measure an animal’s ability to *learn* to wait for rewards, rather than voluntary choices stemming from inherent self-control ability (Pepperberg 2022; Brucks et al. 2023). In spatial discounting tasks, both rewards are visible a set distance apart, so that animals have a visual indication of the extra effort required to obtain the higher-quality option. This reduces the possibility that animals take the immediate lower-quality option simply because they do not know the amount of waiting or effort required for the higher-quality reward (Pearson et al. 2010). To date, spatial discounting tasks have been successfully used with dogs (*Canis lupus familiaris*) (Brady et al. 2018; Mongillo et al. 2019), primates (*Saguinus oedipus* (Stevens et al. 2005b); *Callithrix jacchus* (Stevens et al. 2005b); *Cebus apella nigritus* (Janson 2007)*; Macaca mulatta* (Kralik and Sampson 2012)), guppies (*Poecilia reticulata*) (Mühlhoff et al. 2011), and black garden ants (*Lasius niger*) (Wendt and Czaczkes 2017), suggesting that they are applicable to a range of taxa. However, only two studies have conducted these tasks in the field, both with wild primates (Janson 2007; Kralik and Sampson 2012). Further studies are needed to validate spatial discounting tasks for use with other species in the wild, particularly birds, which remain untested in this paradigm.

Cognitive task performance can be influenced by a range of factors other than the target ability (Rowe and Healy 2014). Examining possible confounds is therefore an important step in validating spatial discounting tasks as a self-control measure. For example, individuals may prioritise instant rewards when they are in poorer body condition and/or less satiated, particularly if the species also has a fast metabolism, as they simply cannot afford to wait or expend limited energy on the better-quality option (Stevens and Stephens 2010). This risk may be exacerbated by the fact that future rewards might never be obtained (Stephens and Anderson 2001; Fawcett et al. 2012). While a captive study found no effect of satiation on spatial self-control decisions in two primate species (Stevens et al. 2005b), this remains to be examined in the field where motivation and satiation are more challenging to standardise. Boldness, a personality trait measuring reactions to risky situations (Réale et al. 2007), may also affect behaviour during self-control tasks, yet has not been examined as a potential confounding factor for any self-control paradigm. Taking the further and/or delayed reward may require the subject to spend more time interacting with the task or travel closer to the experimenter, which could be fear inducing. Less bold individuals may therefore prefer taking immediate, nearer rewards. Finally, the degree of preference an individual has for the higher-quality reward over the lower-quality alternative could affect their choices (Hillemann et al. 2014). Although studies often require individuals to have a defined level of preference to participate in self-control tasks (e.g., preferring one reward over another at least 75% of the time when offered side-by-side (Brucks et al. 2022b)), even minor differences beyond this criterion could affect the amount of time and energy an individual is willing to expend on the better option. Identifying confounds of task performance will enable future experiments to control for these where necessary, improving our ability to isolate and quantify self-control ability through spatial discounting tasks.

In addition to non-cognitive factors, performance during self-control tasks may also be influenced by non-target cognitive abilities (Beran and Hopkins 2018; Schnell et al. 2021, 2022). Accordingly, it has been suggested that inhibitory control, an executive function that allows animals to suppress prepotent responses, may correlate positively with self-control (Amici et al. 2008; Brucks et al. 2017). In particular, motor inhibition, which involves the suppression of prepotent physical actions (Tiego et al. 2018), may be an important building-block for self-control, as subjects must resist the physical impulse to take the immediate reward. Motor inhibition in animals is typically measured using detour tasks, where subjects must inhibit the prepotent motor action of reaching directly for a reward behind a transparent barrier and instead detour to the barrier opening to access the reward (Kabadayi et al. 2018). However, inhibitory control tasks differ from those measuring self-control in that behaving impulsively during inhibitory control tasks is unrewarded (Beran 2015). In contrast, taking the immediate option in self-control tasks is not only rewarded (albeit at a lower value) but can also provide benefits in terms of reduced opportunity and collection risks or maximising intake rate (Stevens and Stephens 2010; Fawcett et al. 2012). This choice component makes self-control more cognitively demanding than inhibitory control (Beran 2015).

There is mixed evidence for relationships between self-control and inhibitory control. Previous studies found no evidence that delay of gratification ability was positively related to performance on inhibitory control tasks in dogs (Brucks et al. 2017) and primates (Völter et al. 2018). In contrast, other studies have found that delay of gratification ability is positively related to performance across a range of cognitive domains in both humans (Boisvert et al. 2013; Meldrum et al. 2017) and animals (Beran and Hopkins 2018; Schnell et al. 2021, 2022), including inhibitory control (Schnell et al. 2021, 2022). To date, however, no study has investigated relationships between self-control in a spatial context and other cognitive abilities. Spatial choices may be driven by factors that do not apply to purely temporal choices, such as the energetic and opportunity cost of locomotion, and risks associated with travel (Mallpress 2021), so performance may not correlate across contexts. For example, marmosets (*Callithrix jacchus*) will delay gratification for longer than tamarins (*Saguinus oedipus*) (Stevens et al. 2005a), but will not travel as far for a better reward (Stevens et al. 2005b). Due to the potential context-specificity of self-control, relationships between temporal and spatial self-control tasks and other cognitive abilities may also differ. It is important to examine relationships between motor inhibition and spatial self-control ability, as although a correlation between these abilities may be expected, it could also confound the interpretation of self-control performance. If an animal performs poorly in both contexts, it may be that they simply could not resist grabbing the first reward they encounter during the self-control task, rather than having attended to the rewards on offer and weighed up the costs and benefits of each.

In this paper, we explored whether wild toutouwai, (*Petroica longipes*), a small songbird endemic to New Zealand, are capable of self-control in a spatial discounting paradigm resembling natural foraging decisions. Additionally, we examined potential cognitive and non-cognitive confounds of task performance to determine whether the spatial discounting paradigm is a valid measure of self-control in this species overall, and whether it reliably captures individual differences in this ability. To achieve this, we presented toutouwai with a spatial discounting task where they decided between a less preferred, near food item or a highly preferred food item placed further away. To assess whether subjects were making informed decisions rather than habitually selecting the furthest option, we used control trials where the near option was of better or equal quality compared to the further reward (Brucks et al. 2022b). To further examine whether the spatial discounting task reliably measured individual differences in self-control, we tested whether a bird’s performance was confounded by body condition, satiation, learning effects, boldness, or the extent of their preference for the high-quality reward. Finally, we tested subjects in a detour task to explore whether individuals with better spatial self-control also showed superior motor inhibition abilities.

## Methods

### General testing information

We tested 20 toutouwai from the long-term research population at Zealandia Ecosanctuary, a 225-ha sanctuary in Wellington, New Zealand (41°18’S, 174°44’E). Toutouwai in this population are free-living and reared entirely in the wild. Small amounts of food are provided to population members during experiments and breeding season monitoring. However, birds are not given additional supplementary food or any other management interventions, and there is no history of captive breeding. Further details on this population are given in McCallum and Shaw (2023). Subjects were 7 female and 13 male colour-banded birds ranging from 6 months to 11 years of age. Testing took place between June and August in 2023 during the toutouwai non-breeding season to minimise seasonal variation in toutouwai behaviour. Birds could only participate in the task if they had a flat area of ground at least 2 × 1 m in a central area of their non-breeding territory, as this was needed to set up the apparatus and ensure that neighbouring conspecifics did not interrupt experiments. We conducted sessions on consecutive calendar days, or the next possible day where this was not feasible (e.g., due to severe weather).

Prior to testing, we conducted pilot sessions with five birds to choose rewards for the self-control task. To be a viable reward system for measuring self-control, we required birds to choose one reward over another in at least 8/10 trials when these were offered side-by-side and they were only allowed to take one option. However, birds also needed to take their less preferred option when it was offered freely, as the low-value reward must be desirable enough that self-control is needed to resist it. Of the five birds we piloted prey size with, only one bird showed a suitably strong preference (reward selected in ≥ 8/10 trials) for a large mealworm (*Tenebrio molitor* larvae) over a small mealworm. Additionally, birds typically refused dried mealworms and tiger worms (*Eisenia fetida*) when offered freely, so these were not pursued as options for the low-quality reward. We therefore used mealworms as the high-value reward and soldier grubs (*Hermetia illucens* larvae) as the low-value reward in the self-control task, as all five subjects strongly preferred mealworms over soldier grubs during pilot sessions (mealworm selected in ≥ 8/10 trials), but still consumed freely available soldier grubs. All rewards were freshly killed at the start of each trial.

During all phases, mealworms were placed on a blue board (10 × 10 cm) and soldier grubs on an orange board (10 × 10 cm) to facilitate reward discrimination. Blue was chosen to be associated with the mealworm, as previous research suggests that toutouwai are attracted to the colour blue (Shaw et al. 2015), and we therefore expected that this would increase the value of the mealworm reward. Orange was chosen for the soldier grub to provide as much contrast as possible with the blue board.

At the end of every session during the preference, training and test phases, birds were offered a single soldier grub on a scale, allowing us to record the bird’s mass to the nearest 0.1 g and check that this low-value reward remained desirable when offered freely. Birds always took this soldier grub. This was crucial to assess – if birds had refused the soldier grub at the end of the session, it would suggest that their ability to resist this reward in previous test trials may have been driven by a perceived lack of value, rather than self-control.

**Fig. 1:**
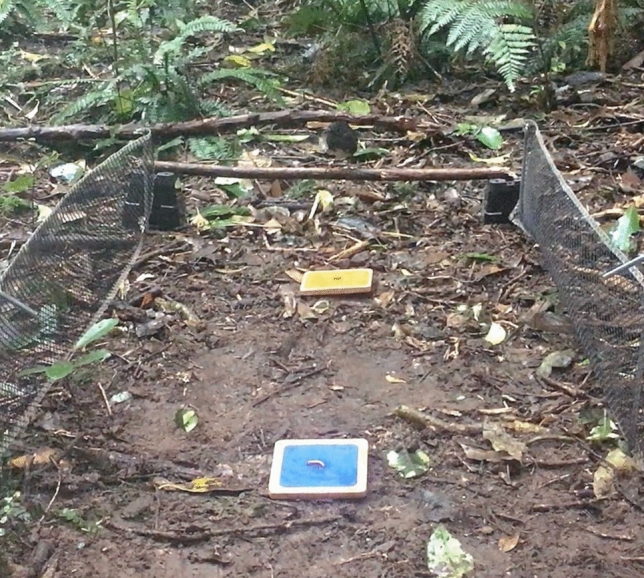
Apparatus set-up showing the position of the nets and perch. Here the boards are shown placed in the positions for a self-control test trial (see Fig. 2 for all board arrangements used), with 50 cm distance to furthest reward shown

**Fig. 2:**
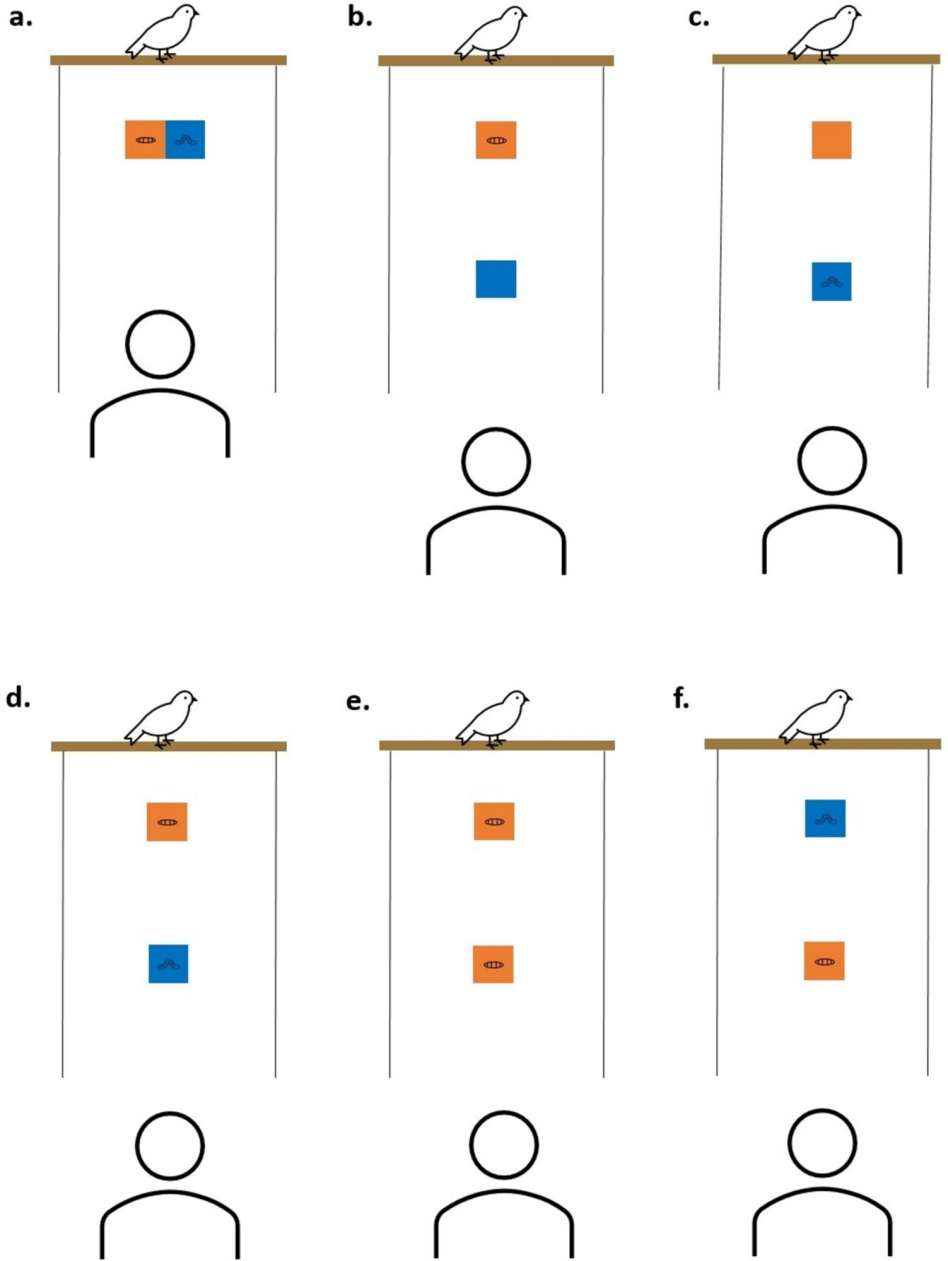
Diagram of the apparatus set-up during a. preference tests, b. training trials with near reward only, c. training trials with far reward only, d. test trials, e. low-quality control trials and f. positional control trials. Mealworm (high-quality reward) is placed on blue board, soldier grub (low-quality reward) is placed on orange board. The experimenter is positioned 80 cm from the furthest board (or both boards in the preference tests)

During the preference, training and test phases, the boards containing rewards were placed between two 150 cm long by 18 cm high plastic nets. These nets were placed 60 cm apart and secured to the ground using metal pegs. The nearest reward (or both rewards during the preference test) was always placed 30 cm from a perch at the start of the nets, created using a natural stick placed on two 9 (W) × 9 (H) × 8.5 (D) cm plastic containers (Fig. 1). During the preference test, this perch provided birds with a starting position as equidistant as possible to the soldier grub and mealworm, minimising the possibility that they simply selected the closest reward. In the training and test phases, the perch ensured that birds first needed to pass the orange board to reach the better-quality reward on the blue board. In most cases birds willingly used this perch to begin a trial. After testing was complete, we assessed perch use from videos for a randomly selected 20% of test trials (*N* = 90), finding that birds started from the perch in 81/90 of these trials. However, birds were permitted to begin a trial from any location (e.g., from surrounding vegetation) provided they approached from the start of the apparatus, approximately where the perch was located, rather than the sides. The experimenter always sat 80 cm from the furthest board (Fig. 2), ensuring that birds did not need to come progressively closer to them to obtain the better-quality reward as the distance between the boards increased. Example trials from the preference test, training phase and test phase are shown in the supplementary video.

### Novel food introduction

All birds had prior experience consuming mealworms through previous experiments and/or routine population monitoring, but none had consumed soldier grubs. To reduce the possibility that birds avoided soldier grubs due to neophobia, on the day before preference testing began, we offered subjects 6 soldier grubs, placed one at a time on the orange board. The first 3 soldier grubs were live so that their movement made them recognisable as prey. The remaining 3 soldier grubs were freshly killed. Birds were allowed a maximum of one minute to take each reward. All birds took each reward within one minute, generally approaching and consuming each reward immediately after was placed on the board.

### Preference test

We conducted preference tests the day after birds completed the novel food introduction. Here, the orange and blue boards were placed side-by-side between the nets (Fig. 2a). Before starting the preference test, we gave birds one free-choice trial where they could take and eat both rewards. This allowed birds to directly compare the two reward types and establish their preference, thus reducing the possibility that they were sampling rewards in the test trials. Birds always consumed both rewards during the free-choice trial. In the following 10 preference test trials, birds could only take one reward. Once the bird retrieved their chosen reward, the experimenter removed the second reward. The position of the boards (left or right) was randomised in each trial by using the ‘RANDBETWEEN’ function in Microsoft Excel to simulate a coin toss. Birds needed to take the mealworm in at least 8/10 test trials to be included in the task. If a subject did not reach this criterion in their first session, they received a second session on the next consecutive testing day, as we expected that birds might initially select their less preferred option first if they expected that both rewards could be obtained during the trial. Twenty of the 21 birds tested in the preference tests reached criterion for inclusion, all achieving this within the first session. One male did not reach criterion after two sessions and was excluded from further testing.

Once birds had completed training and testing for the spatial discounting task (these procedures are described below), they received a second follow-up preference test identical to the test described above. Again, birds received a single trial immediately prior to the preference test where they could sample both rewards, then could only choose one reward per trial in the following 10 preference test trials. The purpose of this second preference test was to assess whether the extent of an individual’s preference for mealworms over soldier grubs had changed once testing was complete, as we expected that increasing familiarity with soldier grubs may make them more desirable. If this was the case, it would raise the possibility that the task became increasingly harder over time not only because of the increasing distance between the rewards, but also because the soldier grub became closer in value to the mealworm and thus harder to resist. One male did not receive his second preference test due to injury.

### Training phase

Training began the following testing day after birds completed the preference tests. Here, the orange board was placed 30 cm from the perch, and blue board was placed a set distance away from the orange board. During a given training trial, either the high or low-value item was placed on its assigned board while the other board remained empty (Fig. 2b, c). Birds received 6 trials with the high-value reward and 6 trials with the low-value reward. One trial with each reward type (high and low value) was given at each of the following distances between the blue and orange boards: 10, 30, 50, 70, 90 or 110 cm. Trial order was randomised between individuals. The training phase taught birds that they could move past the orange board to access the mealworm on the blue board. However, giving trials where only the soldier grub was available ensured that we did not simply train birds to always travel to the furthest board. It also ensured that birds could see the far reward at the distances to be used during the test phase.

To begin a training trial, the experimenter placed the reward on the assigned board and covered each board with a 9 (W) × 9 (H) × 8.5 (D) plastic container attached to an adjustable pole. The position of these reward covers was adjusted along the pole depending on the distance between the boards (Supplementary Fig. 1). Reward covers were crucial during the later test phase to ensure that birds did not grab the first reward placed down before the experimenter had baited the second board and moved into position. As the movement of these reward covers could be fear inducing, we familiarised birds with these during the training phase. Birds could begin a trial once the experimenter had moved into their start position and used the pole to lift and remove the reward covers. All birds retrieved all 12 rewards during their first training session, in almost all cases approaching the reward immediately after the reward covers were removed.

### Test phase

Birds began the test phase the day after completing training. Test sessions consisted of 10 trials in total: 6 test trials (near reward: soldier grub, far reward: mealworm) (Fig. 1, 2d), 2 low-quality control trials (near reward: soldier grub, far reward: soldier grub) (Fig. 2e) and 2 positional control trials (near reward: mealworm, far reward: soldier grub) (Fig. 2f). Each 10-trial test session comprised two, five-trial blocks. Each of these five-trial blocks contained 3 test trials, 1 low-quality control trial and 1 positional control in a randomised order within that block. This was to ensure a relatively even spread of test trials throughout the session, to account for the possibility that the placement of test trials within the session could affect performance. For example, a bird that received all their test trials towards the end of the session may be more satiated by the earlier control trials and thus more likely to pass the test trials.

During control trials, we expected that birds should always select the nearest reward, as this was now of equal to or better quality than the further reward. The purpose of the control trials was to assess whether birds were making informed choices based on the different reward types available and had not simply learned to always travel to the furthest board. The low-quality control trials also allowed us to check that birds were willing to take a soldier grub throughout the session when it was the only reward type on offer.

The setup for test sessions was the same as training, except that both boards now contained rewards (Fig. 2d–f). The order that the experimenter placed down and covered each reward was randomized between trials. Within each session, the near and far rewards remained a set distance from each other. Birds were tested at distances ranging from 10 to 110 cm, increasing at 20 cm increments. If a bird selected the further reward in 4/6 test trials within a session, the distance to the further reward was increased by 20 cm the following testing day. This criterion was chosen as pilot sessions showed that birds rarely chose the far reward in all test trials and thus a stricter criterion would risk a floor effect. Additionally, we kept the number of trials per session low to avoid birds losing motivation. However, this is a relatively strict criterion compared to methodologically similar delay of gratification tasks (Brucks et al. 2022b, a; Schnell et al. 2022). If a bird did not pass this criterion for a given distance in the first test session, they received a second test session at the same distance the next day. If they had not passed by the end of the second session, testing stopped and the previous distance at which they achieved ≥ 4/6 trials (or 0 cm for birds that failed to pass 10 cm) was used as the performance measure.

Birds almost always completed all trials (i.e., took a reward) within a session. However, one female faced interference from her mate. As male toutouwai are dominant to their mates and intercept food rewards, due to his presence we were unable to test her entirely on two days and had to abort one session after she completed 6 trials, 3 of which were failed test trials. As only 2 failed test trials were permitted to pass a session and she had failed that same distance on the previous testing day, we considered her to have failed the task at that distance and did not reattempt the test session. All other birds were tested in isolation from conspecifics.

### Detour task

**Fig. 3:**
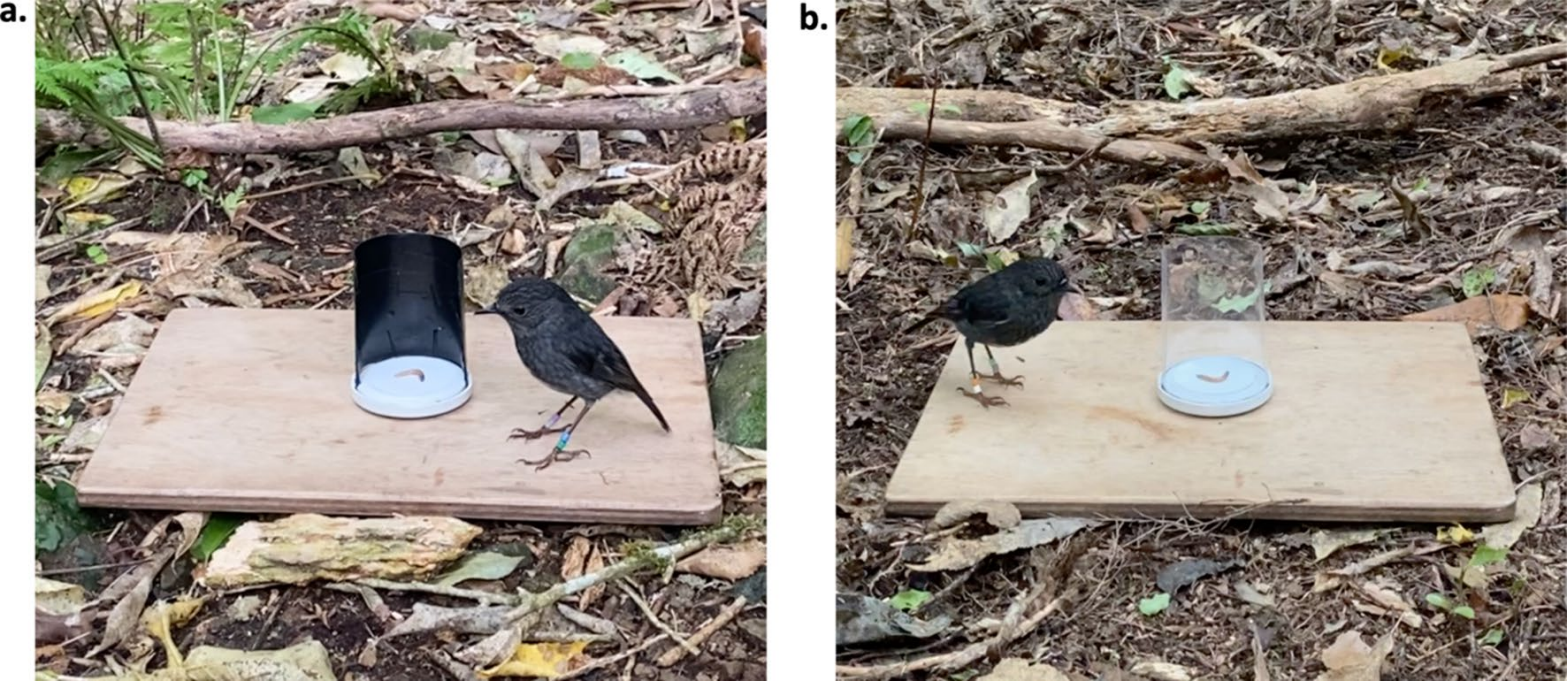
Barriers used during a. habituation and training phases, and b. test phase of the detour task

Following self-control tests, birds received a detour task to assess their motor inhibition abilities. The detour task has been previously validated for use with wild toutouwai, with individual detour task performance showing significant long-term repeatability and resilience to several non-cognitive confounds in this population (McCallum and Shaw 2023). We had tested thirteen of the 20 subjects in an identical detour task the year prior (2022) (McCallum and Shaw 2023). The detour apparatus was a U-shaped plastic barrier (9 cm height × 6 cm diameter) which was opaque during the habituation and training phases (Fig. 3a) and transparent during the test phase (Fig. 3b). Birds received a maximum of 20 trials per day. If a bird did not interact with the task within 2 min in three consecutive trials this was taken as a lack of motivation to participate. In these cases, we ended the session and resumed it on the following testing day. During trials, the experimenter sat approximately 1 m away from the apparatus facing the open side of the barrier.

The detour task began with a habituation phase to reduce neophobia. In the first two trials of habituation, the experimenter placed a mealworm beside the opaque U-shaped barrier (left/right side order randomly selected). In the third trial, the mealworm was placed behind the barrier at the open side. Birds needed to retrieve the worm from each of these locations, each within 2 min, to pass habituation. Birds moved onto the training phase immediately after passing habituation. Here, the worm was placed inside the opaque barrier (Fig. 3a). Birds needed to retrieve the worm from the open side of the barrier within 2 min without first pecking the barrier face in four consecutive trials to pass training.

Birds began the test phase immediately after training was complete. In the test phase, the mealworm was placed behind the transparent barrier (Fig. 3b). To pass a trial, birds needed to detour around the barrier and retrieve the mealworm without first pecking the barrier face within 2 min. Birds needed to pass 6/7 consecutive trials to complete the test, and the number of trials to reach this criterion was the inhibitory control performance measure. All 20 birds completed the detour task.

## Statistical analyses

Data was analysed in R software (R Core Team 2022), using the packages ‘lme4’ (Bates et al. 2015) and ‘glmmTMB’ (Brooks et al. 2017) to run models, and ‘performance’ (Lüdecke et al. 2021) and ‘DHARMA’ (Hartig 2022) to assess model fit.

### Overall task performance

To explore the extent to which toutouwai were capable of self-control in the spatial discounting task, we calculated the range and average maximum distance birds achieved (i.e., chose the far reward in at least 4/6 test trials within two sessions). In this calculation, we only included the 13 birds that achieved the minimum distance of 10 cm and therefore showed that they were capable of self-control.

To evaluate whether birds were attending to the rewards available and had not simply learned to always travel to the further location, we calculated the average percentage of test, low-quality and positional control trials that toutouwai performed successfully per session. Success was defined as selecting the further option during the test trials, and the near option during low-quality and positional control trials. We expected that the percentage of successful trials would be higher in the low-quality and positional control contexts compared to test trials.

### Travel time and reward distance

Spatial discounting tasks require subjects to expend more energy and endure a time delay to reach the further, better-quality reward. To understand the time cost associated with the further reward, we calculated the average travel time between the low-quality and high-quality reward at each of the distances used. Travel time was defined as the time between the bird’s head passing the low-quality reward and their beak touching the high-quality reward, coded from videos by EM using BORIS software (Friard and Gamba 2016). At 10, 30, 50 and 70 cm, travel times were coded for 10 randomly selected, successful test trials per distance. As few birds reached and performed successfully at the furthest distances (only one bird passed 90 cm, and none passed 110 cm), only 9 and 3 trials could be coded at 90 and 110 cm, respectively. In total, 52/218 successful test trials were coded for travel time. RCS independently coded travel time for 50% (26) of these 52 trials to assess interobserver reliability. Interobserver reliability was excellent (ICC = 0.982, *p* < 0.001, 95% CI  = 0.961 to 0.992), so only EM’s travel time estimates were used for analysis. We used a linear mixed model with bird ID and date as random factors to test whether distance between the reward (cm) predicted travel time (s).

### Task validity

To explore whether our spatial discounting task was a valid measure of self-control, we first investigated whether the increasing difficulty of the task could be partly due to soldier grubs increasing in desirability as the birds became more familiar with them. To examine this, we used a paired t-test (*N* = 19) to examine whether the number of mealworms that birds selected during preference tests was lower in the post-task preference test compared to the pre-task preference test.

We further examined task validity by testing which factors affected the outcome of the 452 test trials conducted. We used a generalized linear mixed model with the outcome of each test trial (binary success or failure, with success defined as taking the far reward) as the response variable. As fixed factors, we included the trial number within the session (from 1–10) and the distance (cm) between the near and far rewards. We included bird ID and session date as random factors.

We expected that birds may be more likely to pass a test trial conducted later in the session as they would be satiated by rewards gained during the previous trials, and thus would face less of an impulse to take the first option. Both body condition (assessed in the model described below) and trial number could capture food motivation. However, our body condition measure is a proxy for fat reserves while controlling for skeletal size (Labocha and Hayes 2012), while trial number captures the increase in gut contents, which could be a more relevant predictor of immediate hunger. Additionally, the preceding test trials could remind subjects of the task rules (i.e., that only one reward can be selected) allowing them to perform better during the later trials. We also expected that birds would also be more likely to pass a trial when the distance between the rewards was smaller. However, we note that this analysis almost certainly underestimates the effect of distance on the likelihood of choosing the further reward, as birds were only tested until the distance at which they failed the task. Had we continued to test birds past this point, they likely would have continued to fail trials at greater distances, as has been shown when subjects are tested past the point of failure in temporal self-control tasks (Schnell et al. 2021, 2022). Nonetheless, we included reward distance (cm) in the model also because it was necessary to control for when assessing the effect of trial number on trial outcome.

Finally, we tested which cognitive (inhibitory control) and non-cognitive (body condition, extent of mealworm preference, and boldness) variables predicted a subject’s overall task performance. The inhibitory control measure was the number of trials a bird required to reach criterion in the detour task. We calculated body condition by dividing a subject’s average mass (g) across all sessions of the self-control test phase by their adult tarsus measurement (mm) (Shaw 2017). The degree of each bird’s preference for the mealworm over the solider grub was measured as the total number of trials in which they selected the mealworm across the pre-test and post-test preference tests (maximum 20 trials). We measured boldness towards the task by calculating the average speed (cm/s) at which birds approached the furthest reward in the 6 training trials where only the far reward was available. To take these rewards, birds needed to interact with the task apparatus and approach the board 80 cm from the experimenter’s location, which could be fear inducing. Birds that were faster on average to take the mealworm were thus considered bolder. For each trial, we divided the distance between the starting perch and mealworm by their latency to retrieve the mealworm, coded from videos using BORIS software (Friard and Gamba 2016) as the time between leaving the starting perch and their beak touching the mealworm. In total, EM coded boldness for 91 trials (24 trials could not be coded as the bird either did not start from the perch or the video did not capture the start of a trial). RS independently coded boldness for a randomly selected 20% (*N* = 18) of these trials. Interobserver reliability was excellent (ICC = 0.974, *p* < 0.001, 95% CI  = 0.933 to 0.990), so only EM’s boldness measures were used for analysis. In total, we analysed factors affecting performance for 18 birds. One female was excluded from the model as she could not be located following testing for a tarsus measurement (i.e., either died or dispersed out of the study area), and one male was excluded as he did not receive his second preference test due to injury.

We used a linear model to analyse whether detour task performance (i.e., inhibitory control), body condition, extent of preference for the high-quality reward or boldness predicted overall self-control performance, defined as the maximum distance at which they chose the further reward in at least 4/6 trials during a session (or 0 cm for birds that failed to achieve 10 cm). Square root transformation was applied to self-control scores to ensure normality.

Initially, we expected that prior experience with the detour task in 2022 for 13 test subjects would not confound detour performance, as previous studies found no effect of prior experience in a cylinder detour task on performance in another cylinder detour task (Shaw 2017) or U-barrier detour task (McCallum and Shaw 2023) a year later in toutouwai. However, a negative binomial GLM revealed that experienced birds significantly outperformed naïve birds on the U-detour task in the current study (β ± SE =—0.783 ± 0.141, *p*-value < 0.001, 95% CI = −1.060 to −0.506). This may be because the tasks were identical, whereas the other studies slightly modified the task in their second presentation. To control for this confounding effect, we included whether a bird had prior experience in the 2022 detour task as a binary fixed factor in the linear model described above for factors affecting overall self-control performance.

## Results

### Overall task performance

**Fig. 4:**
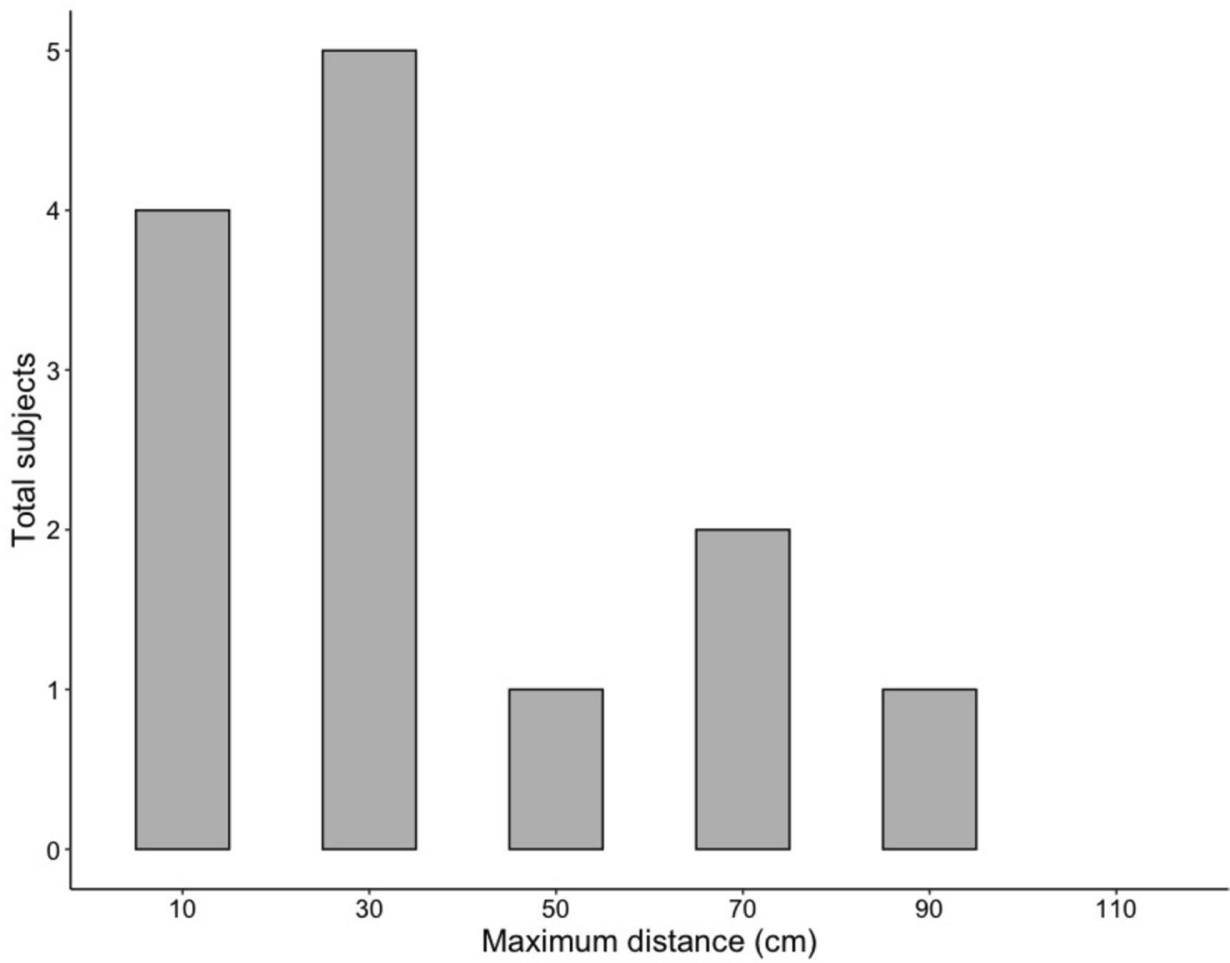
Number of toutouwai that reached each distance during the self-control task (passed ≥ 4/6 test trials within two sessions). 7/20 birds did not pass 10 cm and are not shown here. No birds reached criterion at 110 cm

Toutouwai showed considerable variation in their self-control task performance. All birds (*N* = 20) strongly preferred mealworms over soldier grubs when these were placed side-by-side in the initial preference tests, yet seven birds subsequently failed to reach criterion when the mealworm was 10 cm further away. However, the remaining 13 birds demonstrated that toutouwai are capable of self-control, achieving distances ranging from 10 to 90 cm (Fig. 4). No individual achieved 110 cm. Of the birds that at least passed the minimum distance of 10 cm, the average maximum distance achieved was 36.2 cm (SD = 26.3 cm).

**Fig. 5:**
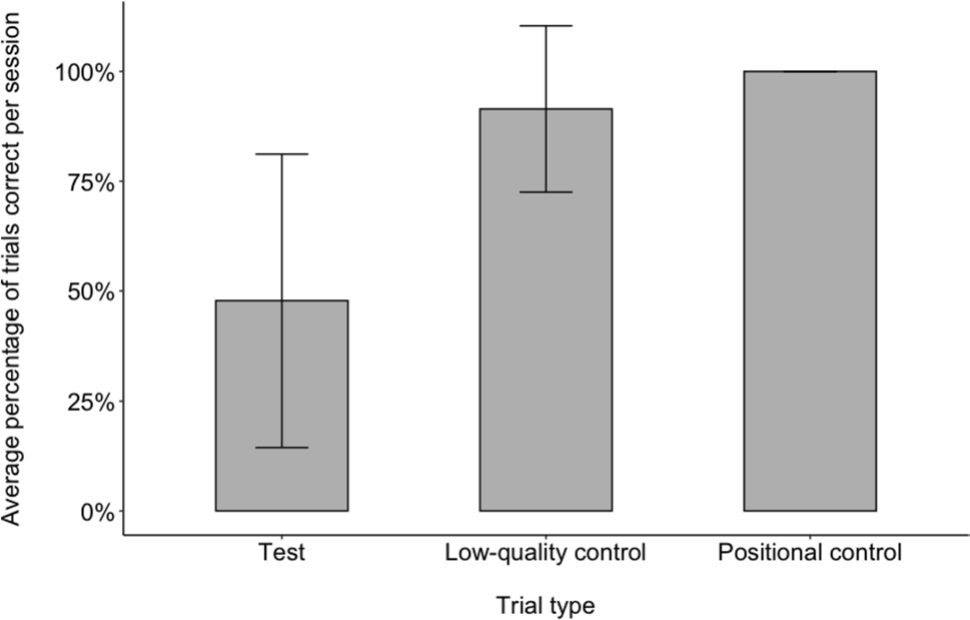
Average percentage of test, low-quality control, and positional control trials toutouwai passed per session. Error bars show standard deviations. Passing defined as choosing far reward in test trials and near reward in control trials. Sessions contained 6 test trials, 2 low-quality control trials and 2 positional control trials

Across all test sessions in this study, on average birds performed successfully on 47.8% (SD = 33.4%) of the six test trials, 91.5% (SD = 19.0%) of the two low-quality control trials, and 100% (SD = 0%) of the two positional control trials in each session (success defined as taking the far reward in test trials and the near reward in control trials) (Fig. 5).

### Travel time and reward distance

During successful test trials, the average travel time between the low-quality and high-quality reward ranged from 0.12 (SD = 0.02) seconds at 10 cm to 2.35 (SD = 0.65) seconds at 110 cm (Table 1). Travel time and distance were positively related (β ± SE = 0.024 ± 0.002, *p* < 0.001, 95% CI = 0.019 to 0.029).

**Table 1:** Average travel time between the low- and high-quality rewards during successful test trials. Where possible, 10 randomly selected test trials were analysed per distance. However, fewer trials were available at 90 cm and 110 cm as toutouwai rarely reached and performed successfully at these distances

Distance between low and high-quality reward (cm)	No. trials analysed	Mean travel time ± SD (seconds)
10	10	0.12 (± 0.02)
30	10	0.83 (± 0.44)
50	10	1.03 (± 0.30)
70	10	1.64 (± 0.58)
90	9	2.15 (± 0.85)
110	3	2.35 (± 0.65)

### Task validity

On average, toutouwai chose the mealworm in 9.4 (SD = 0.8, range = 8 – 10) out of 10 trials during the pre-task preference test, and 8.9 (SD = 0.9, range = 7 – 10) out of 10 trials during the second, post-task preference test. For the 19 birds that received both preference tests, their preference for mealworms over soldier grubs was slightly but significantly lower during the post-task preference test (*t* = 2.799, df = 18, *p* = 0.006).

**Fig. 6:**
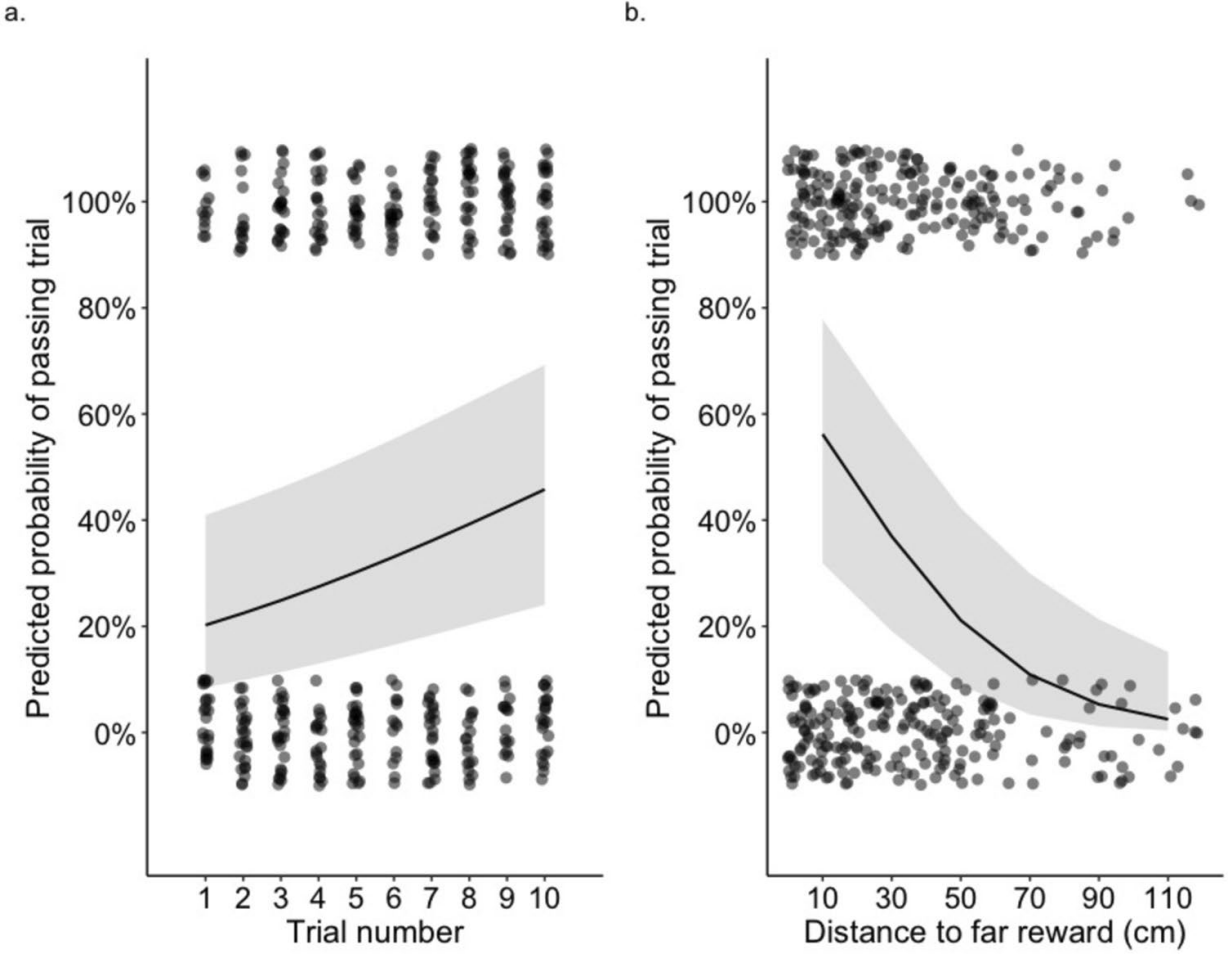
Marginal effects plots showing the influence of a. trial number and b. distance to the far reward on the predicted probability of toutouwai passing a test trial (defined as selecting the far reward). Predicted probability of passing trial shown by trendline. Data points show actual trial outcomes (passed and failed trials shown at top and bottom of graphs, respectively) and have been jittered for visibility

Trial number affected the outcome of test trials, with birds significantly more likely to choose the furthest reward in trials conducted later in the session (β ± SE = 0.134 ± 0.042, 95% CI = 0.050 to 0.217, *p* = 0.005) (Fig. 6a). Additionally, birds were more likely to choose the furthest reward when the distance (cm) to this reward was shorter (β ± SE =−0.039 ± 0.011, 95% CI =−0.061 to−0.018, *p* < 0.001) (Fig. 6b).

**Fig. 7:**
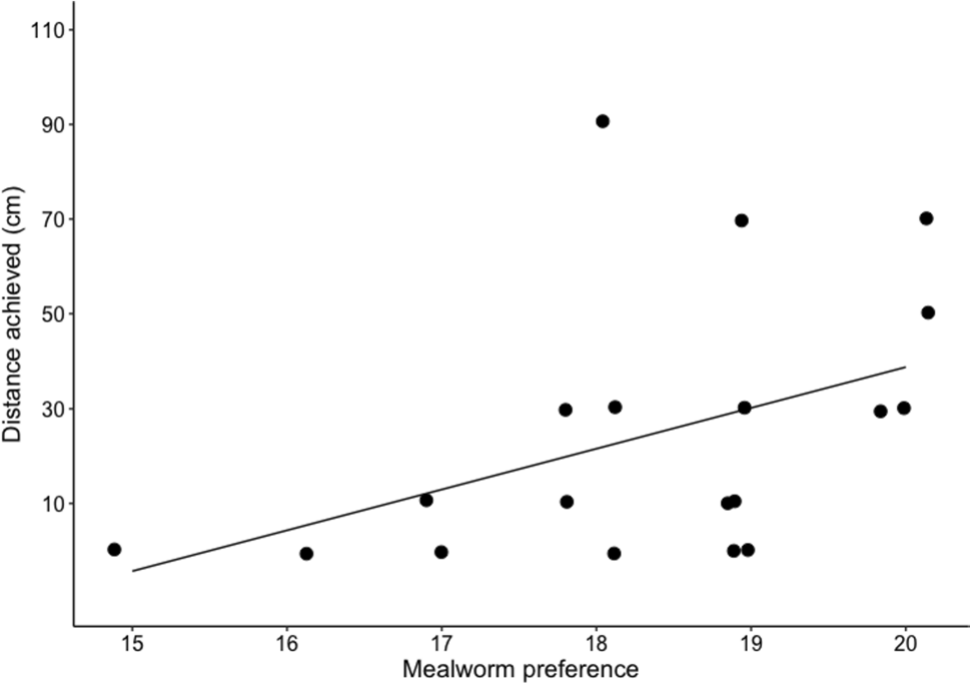
Relationship between the extent of an individual’s preference for mealworms and the total distance they achieved during the test phase. Mealworm preference defined as the total number of trials in which the bird selected the mealworm over the soldier grub in both preference tests. Data points jittered for visibility

A bird’s overall self–control task performance was predicted by the extent of their preference for mealworms over soldier grubs. Birds that selected the mealworm in a greater number of preference test trials achieved significantly greater distances in the self-control task (Fig. 7, Table 2).

**Table 2:** Results of a linear model for factors affecting self-control task performance (square root-transformed maximum distance achieved during test phase; N = 18)

	Estimate	SE	P-value	95% CI
Intercept	15.123	28.372	0.604	−46.696 to 76.942
Extent of mealworm preference	1.312	0.460	0.015	0.309 to 2.315
Previous detour experience	−3.925	1.952	0.067	−8.178 to 0.328
Boldness	−0.058	0.051	0.277	−0.169 to 0.053
Detour score	−0.086	0.224	0.706	−0.574 to 0.401
Body condition	−36.748	34.950	0.313	−112.898 to 39.402

Overall, toutouwai required an average of 11.0 trials to reach criterion (6/7 consecutive trials correct) on the detour task (SD = 3.4, range = 6 to 21). Individuals with prior detour task experience required an average of 7.8 trials to reach criterion (SD = 3.4, range = 6 to 18), compared to 17.0 trials for naïve birds (SD = 4.2, range = 9 to 21). Detour task performance, prior detour task experience, body condition and boldness were not significant predictors of self-control performance (Table 2).

## Discussion

In the spatial discounting task, several wild toutouwai were able to resist consuming a nearer, lower-quality food item in favour of a better-quality option further away. Our results demonstrate that the spatial discounting paradigm is a valid tool for measuring self-control in this species and show for the first time that a bird is capable of self-control in the wild. However, there was striking individual variation in self-control abilities. Seven of our 20 subjects preferred the low-quality reward when a higher-quality alternative was available just 10 cm away, despite strongly preferring the high-quality option when both options were equidistant. In contrast, the remaining 13 birds were able to exert self-control up to an average of 36 cm, with the best performing subject reaching 90 cm. A 10 cm distance translated to a mere 0.1 s of additional travel time on average between the near and far rewards, so it is surprising that a third of subjects preferred the lower-quality reward at this distance. Furthermore, even the longest distance achieved by a toutouwai (90 cm) required a travel time of roughly two seconds. This finding is in keeping with the results of many temporal discounting studies, which show that animals are often unwilling to tolerate delays of more than a few seconds (Rachlin and Green 1972; Ainslie 1974; Ramseyer et al. 2006; Vick et al. 2010). The additional spatial component of this task likely further devalued the higher-quality reward, as toutouwai needed to expend more energy as well as endure a time delay to reach the further option (Mallpress 2021).

Direct comparisons across spatial discounting studies are difficult, as performance is likely influenced by different testing procedures and reward values, and distances must be interpreted relative to an organism’s body size and ease of locomotion. In an impressive display of spatial discounting ability relative to body size, 69% of black garden ants chose a higher-quality food source 60 cm away from a lower-quality alternative (Wendt and Czaczkes 2017). Yet other species seem comparatively intolerant of further rewards. In one study, the percentage of dog’s choices for the higher-value reward dropped from 75% when it was equidistant to the low-value reward, to 35% and 26% when it was 80 and 180 cm further away respectively (Mongillo et al. 2019). In a second study, dogs on average preferred the higher-value reward up to around 300 cm (Brady et al. 2018). Wild rhesus macaques required the quantity of the far reward to be over eight times larger than the near reward for this to become the preferred option when it was 150 cm away (Kralik and Sampson 2012). Researchers have suggested that animals may behave impulsively temporal discounting tasks because the tasks have poor ecological validity and subjects have an incomplete understanding the task rules (Stephens and Anderson 2001; Hayden 2016). Yet with several species, including toutouwai, behaving quite impulsively in spatial discounting tasks that mimic natural foraging decisions, it may be that some animals simply have an inherent preference for immediate rewards.

A species’ preference for immediate rewards may be driven by ecological factors moderating the opportunity and collection risks associated with future options (Fawcett et al. 2012; Mallpress 2021). For instance, toutouwai naturally forage on live, often fast-moving invertebrates (Higgins and Peter 2002), so may strongly devalue further rewards as these may escape before they are reached. However, insectivorous tamarins tolerated longer distances than sap-foraging marmosets in a spatial discounting task (Stevens et al. 2005b), suggesting that specialisation on mobile food-sources may not always lead to impulsive foraging decisions. Another possibility is that toutouwai took the closest reward to progress to the next trial sooner (Smethells and Reilly 2015). While this would not have increased the number of rewards obtained (as only 10 trials were given per session) this would have slightly increased the intake rate. The closely related kakaruwai (*Petroica australis*) spends roughly 90% of its time foraging during winter, suggesting a high metabolic rate in toutouwai (Powlesland 1980). Species with higher metabolic rates are expected to prioritise immediate rewards and fast intake rates, as they are naturally closer their lethal boundary while foraging (Tobin and Logue 1994).

In this study, every toutouwai that we attempted to test participated voluntarily and completed the spatial discounting task. One female toutouwai faced interference from her mate during several trials but still completed testing (see methods). Therefore, it is unlikely that our spatial discounting results were notably influenced by self-selection bias (Webster and Rutz 2020). However, some selection bias was present during the spatial discounting task, as birds were only eligible for inclusion if there was a suitable testing location in their territory. Dominant individuals could occupy larger territories (Candolin and Voigt 2001; Vanpé et al. 2009) which may be more likely to contain a suitable testing location. This may partly explain why males were better represented in our sample, as male toutouwai are dominant to females (Steer and Burns 2008). Dominant individuals may be more tolerant of future rewards as they are better able to prevent competitors from intercepting them (Fawcett et al. 2012; Mallpress 2021). Therefore, it is possible that our results somewhat overestimate the self-control abilities of toutouwai.

Overall, however, our spatial discounting task appeared to be a valid measure of self-control in toutouwai. Subjects virtually always chose the near option when this was of equal or better quality than the far reward. In comparison, they chose the near option in roughly half of test trials, demonstrating that they were attending to the rewards on offer and adjusting their decisions accordingly. Moreover, toutouwai always consumed a soldier grub during low-quality control trials and following each session, indicating that the low-value reward maintained its value throughout sessions. However, the overall preference for mealworms over soldier grubs was slightly but significantly lower during the post-task preference test compared to the pre-task preference test. Although we familiarised birds with soldier grubs before the first preference test, this may not have completely removed neophobia, so birds may have still been somewhat averse to soldier grubs during this preference test. This finding suggests that rewards became more similar in value throughout the task, which would make the immediate reward harder to resist and reduce the level of effort birds would invest in the higher-quality option. Nonetheless, we expect that this did not impact the task’s ability to capture self-control, as the change in preference was minor, and only one individual dropped below a preference of 8/10 choices for the mealworm in the post-task preference test (i.e., the preference criterion required prior to testing to participate in the self-control task).

Self-control likely requires an individual to inhibit the impulse to reach for immediately available food. However, we found no relationship between self-control and detour task performance in toutouwai, which measured an individual’s ability to withhold prepotent motor actions towards visible food. This finding offers further support for this task as a self-control measure, as it suggests that birds who consistently chose immediate rewards were not simply grabbing the first reward encountered rather than attending to the rewards available. The lack of correlation between the tasks may be due to the decision-making aspect of self-control, where subjects must weigh up the costs and benefits of immediate rewards in a given context (Beran 2015). Studies have often found that performance on tasks capturing impulsive behaviours is context specific. In a previous study, we found no positive correlation across a detour task and reversal learning task in this toutouwai population (McCallum and Shaw 2023). Other studies have also found no positive correlation across different forms of inhibitory control (Bray et al. 2014; Brucks et al. 2017; Vernouillet et al. 2018; Szabo et al. 2020; Troisi et al. 2021) or across inhibitory control and delay of gratification tasks (Amici et al. 2008; Brucks et al. 2017; Völter et al. 2018). Thus, it was not wholly unexpected to find no relationship between the detour and self-control tasks. Yet in contrast, other studies have found that delay of gratification ability is positively linked to inhibitory control and a range of other cognitive abilities (Boisvert et al. 2013; Meldrum et al. 2017; Beran and Hopkins 2018; Schnell et al. 2021, 2022). Further research is needed to determine whether delay of gratification ability is linked to inhibitory control in toutouwai, as there is some evidence from primate studies that performance may depend on whether self-control is measured in a temporal or spatial context (Stevens et al. 2005a, b). Furthermore, it remains unknown how spatial discounting ability relates to other cognitive traits.

Associative learning may correlate positively with self-control task performance, as subjects must learn that taking their less preferred option will prevent them from obtaining the higher-quality alternative in that trial. Our preference tests and training phase attempted to teach birds that only one reward could be obtained per trial, and that they needed to pass the first board to access their preferred reward. However, some birds may have performed poorly as they did not fully understand this rule. Birds were more likely to pass a trial conducted later during a session, which may have been partially driven by the reinforcement of the single-choice selection rule during the preceding trials. Striking a balance between ensuring that subjects understand the task rules, whilst maintaining the task’s ability to capture natural behavioural responses, is an ongoing challenge in self-control research (Pepperberg 2022; Brucks et al. 2023).

Robustly measuring individual differences in self-control is crucial to understand how selection operates on this ability and how individual variation in self-control relates to variation in other cognitive and non-cognitive traits. While our spatial discounting task appears to be a valid paradigm for measuring self-control in toutouwai overall, non-cognitive variables confounded its ability to capture individual differences in this ability. Boldness and body condition had no effect on an individual’s self-control performance, but birds with a stronger preference for mealworms were willing to travel significantly further for this reward. This finding was somewhat surprising, as there was relatively little variability in the extent of birds’ preferences, with the number of trials in which individuals selected the mealworm over the soldier grub ranging from just 15 to 20 across both preference tests. Animals should be willing to travel further as the desirability of the far reward relative to the near option increases, so this finding does further validate the task as a measure of self-control overall. However, it confounds the interpretation of individual performance, as a bird with a stronger preference for mealworms over soldier grubs would require less self-control to pass a given distance. Ensuring consistency of reward valuation across time and individuals is likely to be challenging, as this could be subject to personal taste preferences (Liem and Russell 2019), reproductive state (Verbeke and De Bourdeaudhuij 2007), sensory specific satiety (Rogers et al. 2021), or neophobia (Costa et al. 2020). A potential solution could be to use rewards differing in quantity or size rather than quality, as these should maintain a fixed value. While we did not find a sufficiently strong preference for a large mealworm over a small mealworm during pilot sessions (see methods), increasing the size discrepancy could make this a viable option for self-control testing. Additionally, while we did not pilot the use of quantity in the current study, toutouwai have been shown to discriminate between varying prey quantities (Garland et al. 2012). However, as satiation emerged as a likely driver of task performance (see below) this may not be a viable option for studies focussed on individual differences in self-control.

Although we found no effect of body condition on overall performance, birds were more likely to pass trials conducted later in the test session, suggesting that food motivation in terms of gut contents may influence an individual’s self-control performance. As birds became satiated by rewards gained during the earlier trials, the immediate option may have become less tempting. Additionally, as mentioned above, the preceding test and control trials may have reminded birds of the task rules (i.e., that only one reward can be obtained per trial), allowing them to perform better in the later trials. A study of two primate species found a similar tendency for subjects to select the furthest reward in later trials during a session, although this was not significant (Stevens et al. 2005b). Studies focussed on individual differences in self-control could manage this trial number effect by ensuring that control and test trials are in a consistent order across individuals and sessions. While full control of satiation is only feasible in captivity where food-intake prior to testing can be regulated, field experiments could increase the inter-trial interval (e.g., conduct one trial per day) to avoid subjects becoming satiated by previous trials. In a species like toutouwai that caches surplus food, it may also be possible to standardise satiation by pre-feeding subjects until the onset of caching.

Finally, future research into the validity of spatial discounting tasks as a measure of individual differences in self-control should investigate whether task performance is temporally repeatable. Repeatability describes the proportion of variance attributable to differences between individuals (Nakagawa and Schielzeth 2010), and substantial repeatability in performance provides evidence that a cognitive task reliably measures consistent individual variation in the target ability (Wilson 2018; Cauchoix et al. 2018). Additionally, measuring repeatability is a valuable step in exploring the evolution of self-control, as repeatability sets the upper limit to trait heritability (Falconer and Mackay 1996). While a previous study found high individual consistency in adult dogs’ spatial discounting performance across a 4–6 week interval (Brady et al. 2018), the repeatability of performance in spatial discounting and other self-control tasks in the wild, and across longer timescales, is currently unknown.

## Conclusions

Spatial discounting tasks offer an ecologically relevant context for measuring self-control, yet only two previous studies with primates have used these tasks in the wild (Janson 2007; Kralik and Sampson 2012). Wild toutouwai showed considerable individual variation in the distances they were willing to travel for a higher-quality reward, and demonstrated for the first time that birds can exert self-control in a spatial context. This task was designed to replicate natural foraging conditions. Nonetheless, some individuals remained impulsive throughout testing, supporting the possibility that the poor self-control often observed in animals may not be solely due to a lack of ecological validity in task design. In a species like toutouwai with a fast metabolism and specialisation on mobile prey, prioritising immediate rewards over more distant options may be an adaptive choice. We found no relationship between motor inhibition in a detour task and self-control performance, further suggesting that birds did not choose nearer rewards solely because they could not inhibit the impulsive action of reaching towards immediate, visible food. However, the task was also susceptible to some extraneous variables – namely, interindividual differences in reward valuation and the satiation and/or learning effects associated with increasing trial number. Procedural adjustments will likely be needed to reliably measure individual differences in self-control using spatial discounting tasks in toutouwai and other species. Nonetheless, this study provides a valuable starting point in the design of ecologically and methodologically valid measures of self-control in the wild, which will enable us to answer important questions about the evolution of this cognitive ability.

## Supplementary Information

Below is the link to the electronic supplementary material.

Supplementary file1 (DOCX 1301 KB)

Supplementary file2 (MP4 135351 KB)

## Data Availability

All data and R code associated with this study is available on Figshare, https://doi.org/10.6084/m9.figshare.c.7404898
