# Supplementary material for: Measuring self-control in a wild songbird using a spatial discounting task
Source: Anim Cogn. 2024 Oct 25;27(1):70. doi: 10.1007/s10071-024-01911-4 (PMC11511709; doi:10.1007/s10071-024-01911-4)
Supplement: Supplementary file 1 — Supplementary file1 (DOCX 1301 KB) [file 10071_2024_1911_MOESM1_ESM.docx]

**Supplementary material**

**Measuring self-control in a wild songbird using a spatial discounting task**

**Authors**

Ella McCallum^1^ & Rachael C. Shaw^1^

**Affiliations**

1. School of Biological Sciences, Te Herenga Waka Victoria University of Wellington, Wellington, New Zealand

**Corresponding author**

Ella McCallum: ella-mccallum@outlook.com


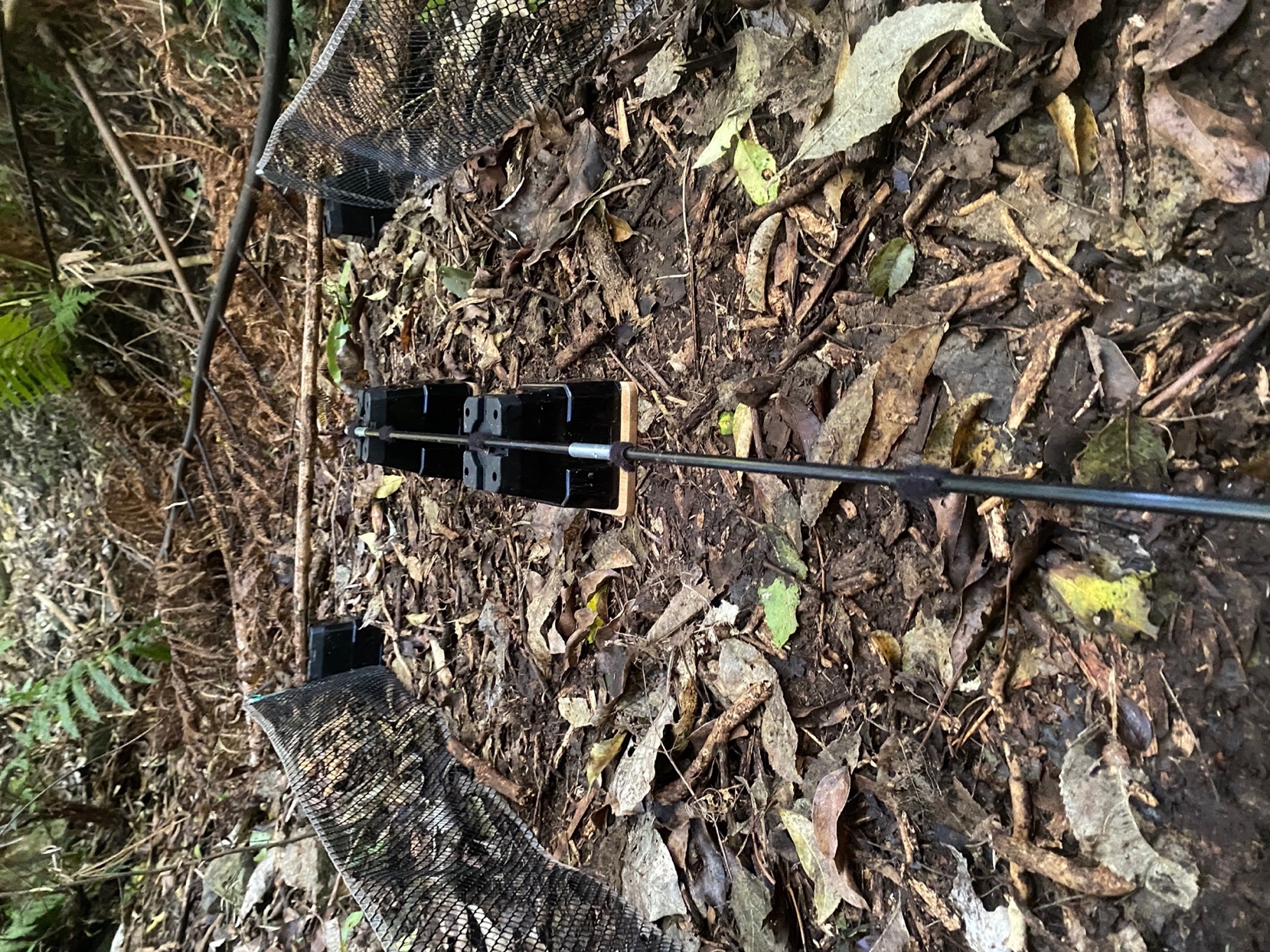


**Supplementary Fig. 1.** Reward covers used during training and test phases to prevent toutouwai from accessing rewards until a trial began. Position of reward covers adjusted depending on the distance between the boards (30 cm shown here).
